# Nearly half of preschool children are stunted in Dembia district, Northwest Ethiopia: a community based cross-sectional study

**DOI:** 10.1186/s13690-016-0126-z

**Published:** 2016-04-18

**Authors:** Amare Tariku, Haile Woldie, Abel Fekadu, Akilew Awoke Adane, Ayanaw Tsega Ferede, Segenet Yitayew

**Affiliations:** Department of Human Nutrition, Institute of Public Health, College of Medicine and Health Sciences, University of Gondar, Gondar, Ethiopia; Department of Epidemiology and Biostatistics, Institute of Public Health, College of Medicine and Health Sciences, University of Gondar, Gondar, Ethiopia; Department of Optometry, School of Medicine, College of Medicine and Health Sciences, University of Gondar, Gondar, Ethiopia; North Gondar Zonal Health Department, Planing, Monitoring, and Evaluation Officer, Amhara, Ethiopia

**Keywords:** Northwest Ethiopia, Preschool children, Stunting

## Abstract

**Background:**

Stunting has been the most pressing public health problem throughout the developing countries. It is the major causes of child mortality and global disease burden, where 80 % of this burden is found in developing countries. In the future, stunting alone would result in 22 % of loss in adult income. About 40 % of children under five-years were stunted in Ethiopia. In the country, about 28 % of child mortality is related to undernutrition. Thus, the aim of this study was to determine the prevalence and determinants of stunting among preschool children in Dembia district, Northwest Ethiopia.

**Methods:**

A community based cross–sectional study was carried out in Dembia district, Northwest Ethiopia from January 01 to February 29, 2015. A multi-stage sampling followed by a systematic sampling technique was employed to reach 681 mother-child pairs. A pretested and structured questionnaire was used to collect data. After exporting anthropometric data to ENA/SMART software version 2012, nutritional status (stunting) of a child was determined using the WHO Multicenter Growth Reference Standard. In binary logistic regression, a multivariable analysis was carried out to identify determinants of stunting. The Adjusted Odds Ratio (AOR) with a 95 % confidence interval was computed to assess the strength of the association, and variables with a *P*-value of <0.05 in multivariable analysis were considered as statistically significant.

**Results:**

A total 681 of mother-child pairs were included in the study. The overall prevalence of stunting was 46 % [95 % CI: 38.7, 53.3 %]. In multivariable analysis, the odds of stunting was higher among children whose families had no latrine [AOR = 1.6, 95 % CI: 1.1, 2.2)]. Likewise, children living in household with more than four family size [AOR =1.4, 95 % CI: 1.1, 1.9)] were more likely to be stunted.

**Conclusions:**

This study confirms that stunting is a very high public health problem in Dembia district. The family size and latrine availability were significantly associated with stunting. Hence, emphasis should be given to improve the latrine coverage and utilization of family planning in the district.

## Background

Stunting, chronic undernutrition, is resulted from long-term exposure to restricted nutrient supply and frequent infection [1]. Though changes in eating patterns and lifestyles, and economic development have contributed to decline in rates of childhood stunting in the world [1, 2], it has been the most pressing public health problem throughout the developing countries [3–5]. According to the recent global estimates, 165 million (26 %) children under 5 years are stunted [6]. More than 90 % of the world’s stunted children are living in Africa and Asia, with the prevalence of 36 % and 27 %, respectively [7].

Childhood stunting is associated with poor cognition and school performance [8, 9]. Besides to this, it poses adverse functional consequences during adolescent and adulthood period, such as low adult wages, lost productivity, and overweight, obesity, and nutrition-related chronic diseases [3, 8]. Particularly, stunting alone would result in 22 % of loss in adult income, however its impact worsen when it is coupled with poverty, which causes 30.1 % of loss in adult income [7]. Furthermore, stunting is one of the major causes of child mortality and global disease burden, where 80 % of the burden is found in developing countries [10].

The government of Ethiopia has implemented a comprehensive nutritional programs over the past decades to improve the nutritional status of children [11, 12]. Accordingly, the country has made substantial improvements in reduction of the burden of childhood stunting. However, about 40 % of children under five–years were still stunted [13], which conformed a very high public health significance when compared with the WHO threshold level [14]. Ethiopia also exhibited the highest burden of child mortality and morbidity [15], of which about 28 % of this mortality is related to undernutrition. In addition, sixteen percent of all repetitions in primary school are also associated with stunting [16].

Child growth is the result of complex and interwoven factors which mainly related to the socioeconomic, health, and dietary habit related characteristics of children [10, 13, 17, 18]. Among the factors which were commonly reported by different studies; mother’s nutritional status, child’s sex, parental education, place of residence, child caring practice, access to health care, latrine availability, and source of drinking water were significantly associated with stunting [19–23]. Particularly, the odds of stunting was higher among children whose mothers were illiterate [20, 24, 25]. Likewise, the likelihood of stunting was higher among children whose parents were in the lowest socio-economic status [26–28].

Hence, showing the magnitude of stunting will have vital importance to address the adverse consequences of stunting among children. Determining the magnitude of stunting among preschool children, the new entrants of school in the near future, might provide a baseline evidence for better estimation of functional consequences, including poor cognition, however, most of the studies in the country merely focused on children aged 6–59 months [10, 29–31]. Thus, the study aimed to assess the prevalence and determinants of stunting among preschool children (24–59 months) in Dembia district.

## Methods

### Study design and setting

A community-based cross-sectional study was conducted from January 01 to February 29, 2015 in Dembia district, Northwest Ethiopia. The district has 45 kebeles *(smallest administrative units),* of which 40 are rural. A total of 270,994 people lived the district [32]. As per the 2015 district health office report, 18,006 preschoolers also lived in the district, and ten Health Centers and forty Health Posts provide health service to the community ***.*** Surrounded by the great Lake Tana, the district is a well-known malaria endemic area. The residents are by and large subsistence farmers cultivating mainly cereals, legumes, and spices.

### Sample size, sampling procedure, and study participants

Preschool children who lived in the district for at least six months were included in the study. The minimum sample size was determined using the formula to estimate single population proportion with the following assumptions: the expected prevalence of stunting as 50 %, a 95 % confidence level, and 5 % margin of error (d). Finally, a minimum sample size of 692 was obtained after anticipating a 20 % non-response rate and adjusting design effect of 1.5. A multi-stage sampling followed by a systematic sampling technique was employed to select the study subjects. Initially, nine representative kebeles in the district (1 urban and 8 rural) were selected using the lottery. The total number of preschool children (3477) living in the selected kebeles was obtained from the district health office and used to calculate the sampling fraction (k). After a proportional allocation to each kebele, the systematic sampling technique was employed. In those households with more than one eligible study subject, lottery was used to select only one child. When mother-child pairs were not available at the time of data collection, two repeated visits were made.

### Data collection instruments and procedure

Data were collected through a face-to-face interview by using a pretested and structured questionnaire. The questionnaire consisted of socio-demographic and economic characteristics, health, and feeding pattern related information. To maintain its consistency, the questionnaire was first translated from English to Amharic, the native language of the study area, and was retranslated to English by professional translators (English language expertise). Two experienced public health experts and 12 trained data collectors (2 Public health officers and 10 clinical nurses) were recruited for supervision and data collection, respectively. The investigators coordinated the overall activities of data collection. The tool was piloted on 5 % of the sample size outside the study area. During the pre-test, the acceptability and applicability of the procedures and tools were evaluated. Household wealth index was computed using a composite indicator for urban and rural residents by considering properties like, livestock ownership, selected household assets, size of agricultural land, and the quantity of crop production. Principal component analysis (PCA) was performed to categorize the household living standards into lowest, middle, and highest.

### Anthropometric measurement

Child weight was measured to the nearest 0.1 kg by the seca beam balance (German, Serial No. 5755086138219) with graduation of 0.1 kg and a measuring range of up to 25 kg. Weight was taken with light clothing and no shoes. Instrument calibration was carried out before weighing each child. Furthermore, the weighing scale was checked against a standard weight for its accuracy on a daily basis.

Height was measured using the seca vertical height scale (German, serial No. 0123) standing upright in the middle of the board. The child’s head, shoulders, buttocks, knees, and heels touch the vertical board. Most of study participants’ birth date was extracted from the Immunization status certificate (immunization card) of the child. However, for nine study subjects, their age was determined based on the information given by the mother/caretaker of the child.

Anthropometric related data of a child were transferred to the ENA/SMART software version 2012 and the Z-scores of indices, Height-for-Age Z-scores (HAZ), was calculated using the WHO Multicenter Growth Reference Standard**.** The child was classified as stunted if his/her z score was less than −2SD; otherwise, he/she was well-nourished (≥ − 2 Z score) [33].

### Assessment of dietary diversity

In Dembia district, there was low intra individual variability regarding the dietary pattern of children. Accordingly, prolonging the reference period in capturing the dietary habit of the study participants may not bring a substantial difference. Thus, Dietary Diversity Score (DDS) of the child was determined using 24-hours recall method. Mothers were asked to list all food item consumed by the child in the previous 24 hours preceding the survey. In case of mixed dish, the ingredients of the food items were listed by the mother. Then, reported food items were classified into seven food groups, as starchy staples (grains, roots, and tubers); legumes, nuts and seeds; vitamin-A rich fruits and vegetables; other fruits and vegetables; egg; dairy products (milk, yoghurt, and cheese); and flesh foods (meat, fish, poultry, and organ meats) [34]. Considering four food groups as the minimum acceptable dietary diversity, a child with a DDS of less than four was classified as poor dietary diversity.

### Data processing and analysis

Data were entered into EPI-INFO version 3.5.3 and analyzed using the Statistical Package for Social Sciences (SPSS) version 20. Descriptive statistics, including frequencies and proportions was used to summarize the study variables. A binary logistic regression was fitted, variables with a *p*-values of < 0.2 in the bivariable analysis were entered in the multivariable analysis to control the possible effect of confounders. In multivariable analysis, backward selection method was used to identify factors associated with stunting. The Adjusted Odds Ratio (AOR) with a 95 % confidence interval was estimated to assess the strength of association, and a *p*-value of < 0.05 was used to declare the statistical significance in the multivariable analysis.

### Ethical considerations

Ethical clearance was obtained from the Institutional Review Boards of the University of Gondar. An official permission letter was secured from Dembia District Health Office. All mothers or caretakers of children were informed about the purpose of the study, and interview was held only with those who agreed to give a written consent to participate. Uneducated mothers affirmed their consent by their thumbprint. The right of a participant to withdraw from the study at any time, without any precondition was disclosed unequivocally. Moreover, the confidentiality of information obtained was guaranteed by all data collectors and investigators by using code numbers rather than personal identifiers and by keeping the questionnaire locked.

## Results

### Socio-demographic and economic characteristics of study participants

A total of 681 mother-child pairs were included in the study, making a response rate of 98.4 %. The mean age (±SD) of the children was 41.58 months (±11.27), and slightly more than half (53.6 %) of them were male. Almost all (93.1 %) of the participants were living in the rural kebeles of Dembia district. In the study area, nearly one-third (30 %) of the households (HHDs) had ≥7 family members. The majorities (95.4 %) of the mothers were housewives, uneducated (77.1 %), and gave their first birth before the age of 20 (63.1 %) (Table 1).

**Table 1:** Socio-demographic and economic characteristics of study participants in Dembia District, Northwest Ethiopia, 2015

Characteristics	Frequency	Percent (%)
Child age in (months)		
24-36	288	42.3
37-48	233	34.2
49-59	160	23.5
Sex of child		
Male	365	53.6
Female	316	46.4
Residence		
Urban	47	6.9
Rural	634	93.1
Marital status		
Single	25	3.7
Married	613	90
Others^a^	43	6.3
Religion		
Orthodox Christianity	674	99
Others^b^	7	1
Ethnicity		
Amhara	673	98.8
Others^f^	8	1.2
Household size		
≤4	226	33.2
5-6	251	36.8
≥7	204	30.0
No. of children ever born		
≤2	195	28.6
3-5	355	52.1
≥6	131	19.2
Birth order of a child		
1^st^	128	18.8
2^nd^-4^th^	362	53.2
≥5^th^	191	28
Maternal education		
Uneducated	525	77.1
Primary	58	8.5
Secondary and above	98	14.4
Maternal employment status		
Housewife	650	95.4
Others^c^	31	4.6
Mothers age		
15-34	495	72.7
35-49	186	27.3
Mothers age at first birth		
≤19	430	63.1
20-49	251	36.9
Paternal education		
Uneducated	410	60.2
Educated	271	39.8
Paternal employment		
Farmer	620	91
Merchant	32	4.7
Others^d^	29	4.3
Wealth index		
Poor	226	33.2
Middle	228	33.5
High	227	33.3
Main source of family food		
Own production	575	84.4
Other^e^	106	15.6

^a^Divorced, widowed and separated

^b^Muslim and protestant

^c^Merchant, government employer and student

^d^Government employer and daily laborer

^e^Purchasing and family assistant

^f^-Oromo and Tigre

### Health and nutrition related characteristics of study participants

**Fig. 1:**
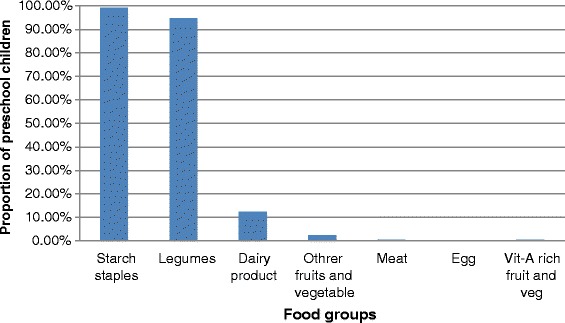
Proportion of preschool children who consumed food groups in the previous 24-h preceding the survey, Dembia district, 2015

The majority (85.8 %) of the mothers had at least one Antenatal Care (ANC) visit for the index child, but around one third (29.9 %) of them gave birth at heath facilities. Most (82.5 %) of the children had a dietary diversity score of below four. Nearly three-fourths (70.3 %) of the mothers initiated breast feeding timely, within an hour of delivery, and a significant proportion of the mothers (65.9 %) initiated complementary feeding at the six^th^ month (Table 2). The dietary pattern of children in the district mainly depends on starchy staples (99.3 %) and legumes (94.9 %), but only few percentage (0.3 %, 0.4 %, and 0.6 %, respectively) of children ate egg, vitamin-A rich fruits or vegetables, and meat in the previous 24-hours preceding the date of survey (Fig. 1).

**Table 2:** Health and nutrition related characteristics of study participants in Dembia District, Northwest Ethiopia, 2015

Characteristics	Frequency	Percent
ANC visit		
Yes	584	85.8
No	97	14.2
Place of delivery		
Home	480	70.5
Health facility	201	29.5
Discarding of colostrums		
Yes	344	50.5
No	337	49.5
Initiation of breast feeding		
Early initiation	479	70.3
Late initiation	202	29.7
Pre-lacteal feeding		
Yes	344	50.5
No	337	49.5
Complementary food initiation		
Timely	449	65.9
Early	33	4.8
Late	199	29.2
Dietary diversity score		
<4 food groups	562	82.5
≥4 food groups	119	17.5
Immunization status		
Partially immunized	121	17.8
Fully immunized	560	82.2
Any morbidity in the last 2 week		
Yes	108	15.9
No	573	84.1
Source of drinking water		
Unprotected source	67	9.8
Protected source	614	90.2
Time to fetch drinking water		
≤30 min	592	86.9
>30 min	89	13.1
Availability of solid west disposal		
Yes	341	50.1
No	340	49.9
Availability of liquid waste disposal		
Yes	328	48.2
No	353	51.8
Availability of latrine		
Yes	531	78
No	150	22

### Prevalence of stunting among preschool children

**Fig. 2:**
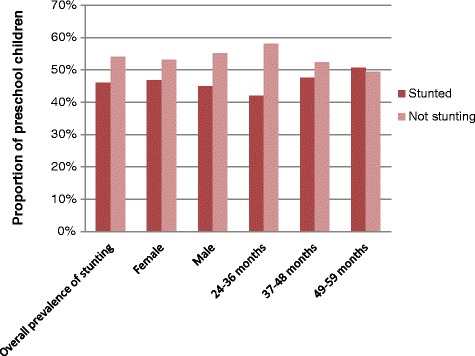
Prevalence of stunting by sex and age category among preschool children in Dembia district, 2015

The overall prevalence of stunting in the district was 46 % [95 % CI: 38.7, 53.3 %], of which about 49.8 % of children were severely stunted (Fig. 2).

### Determinant factors of stunting among preschool children

In bivariable logistic regression analysis, the family size, source food, and latrine availability were significantly associated with stunting. However, in the multivariable logistic regression analysis, household size and latrine availability remained significantly and independently associated with stunting. Accordingly, the likelihood of stunting among children whose family had no latrine was 60 % [AOR = 1.6, 95 % CI: 1.1, 2.2)] more as compared to their counterparts who had latrine. Likewise, the odds of stunting was 40 % [AOR =1.4, 95 % CI: 1.1, 1.92)] higher among children whose parents had a family size of more than four compared with children whose parents had a family size of less or equal to four (Table 3).

**Table 3:** Determinant factors of stunting among preschool children in Dembia district, Northwest Ethiopia, 2015

Characteristics	Stunting			
	Yes#	No#	COR (95 % CI)	AOR (95 % CI)
Discarding colostrum				
Yes	167	177	1	
No	146	191	0.8 (0.6, 1.1)	
Household size				
≤4	94	132	1	1
>4	219	236	1.3 (1.01, 1.8)	1.4 (1.1, 1.9)*
Main source of household food				
Own production	258	317	1	
Other	55	51	1.3 (1.2, 2.1)	
Complementary food initiation				
Timely	217	232	1	
Early	13	20	0.7 (0.3, 1.4)	
Late	83	116	0.8 (0.6. 1.1)	
Mothers age at first Birth				
15-19 years	205	225	0.8 (0.6, 1.1)	
20-39	108	143	1	
Age of child in month				
24-36	121	167	1	
37-48	111	122	1.3 (0.9, 1.8)	
49-59	81	79	1.4 (0.96, 2.0)	
Latrine availability				
Available	233	298	1	1
Not available	80	70	1.4 (1.0, 2.1)	1.6 (1.1, 2.2)*
Dietary diversity				
<4	262	300	1.2 (0.9, 1.7)	
≥4	51	68	1	

Note: *Significant at *p*-value < 0.05

## Discussion

Assessment of growth is the single measurement that best defines the nutritional and health status of children, and provides an indirect measurement of the quality of life for the entire population [35]. Stunting measures cumulative deficient of growth associated with the long-term factors, including insufficient dietary intake, frequent infections, poor feeding practices over a sustained period of time, and low socioeconomic status of the households [36]. Thus, this study assessed the prevalence and determinants of stunting among preschool children.

The prevalence of stunting was high (46 %) in the district, and confirms very high public health significance [14]. The result was consistent with the mini-Ethiopian Demographic and Health Survey report (40 %) [13], and the average estimate of stunting for developing countries (42.7 %) [37]. Likewise, the finding was in agreement with the study reports of other developing countries, such as India (43 %) [38], Nigeria (44.9 %) [39], and Bangladesh (39.5 %) [40]. This is probably due to contextual similarities in socio- demographic and economic characteristics, and feeding pattern of children of the study areas.

However, the prevalence of stunting was highest compared with the study findings in Somali, Ethiopia [30], Ghana (27 %) [41], China (27 %) [42], and Iran (11.5 %) [43]. This discrepancy could be attributed to the difference in age of children included in the studies, in which the latter studies included children less than 24 months while only children aged 24–59 months were included in the current study. However, children found in this age category were less likely to be stunted compared with children aged beyond 24 months [41, 44]. Hence, the study included the older children; the magnitude of stunting was overestimated. In contrast to the latter abroad studies, most of the mothers were illiterate and housewife in the study area. The low maternal educational status was associated with higher odds of childhood stunting [15, 39, 40, 45]. Moreover, low educational status, particularly among household heads negatively affects the household food security status [46]. Though housewife mothers had better time to care their child, in the context of Ethiopia, most of them were not engaged in productive work compared with mothers in other employment status. However, almost all of the mothers were housewives in the study area, of which more than three-fourth of them were illiterate.

The result of the study also demonstrated that, large family size increases odds of having stunting in preschool children. Similar findings were reported in different studies [47–51]. This might be related to the complex interaction between the household food security status, family size, and food consumption pattern of children. As it was revealed by other studies, large family size negatively affects the household food security status mainly through increasing the food expenditure per capita, thereby posing difficulties in securing the per-capita food availability in larger households [46, 52]. Food insecurity hardly affects the household food consumption pattern though forcing them to shift in purchasing low quality food, skip meal, and relay on monotonous diet. However, such poor dietary habits [53], and poor household food security status were associated with stunted growth [54–56].

The likelihood of stunting among children whose families had no latrine was higher as compared to those who had. This finding was concurrent with the findings reported from developing countries [57–60]. This could be related to the importance of latrine availability in promoting optimal hygiene and sanitation in the household and the community at large. Improved hygiene and sanitation is found with reduced risk of childhood stunting, which mainly operates through reducing risk of recurrent diarrhea and other gastro-intestinal related infections [61, 62].

Some of the limitations of this study should be noted and taken into consideration. First, since the study utilized a cross-sectional study design, findings could not show the casual relationship between stunting and other independent variables. Second, there is a potential recall bias among respondents while answering questions related to events happened in the past, such as child feeding practices. Nevertheless, maternal nutritional conditions were potential confounders of stunting in children, the study did not gathered information. Lastly, we didn’t capture information regarding the utilization of latrine.

## Conclusions

This study confirms that stunting is a very high public health problem in Dembia district. The household family size and latrine availability were significantly associated with stunting. Hence, emphasis should be given to improve the latrine coverage and utilization of family planning in the district.
